# Meropenem extraction by *ex vivo* extracorporeal life support circuits

**DOI:** 10.1051/ject/2023035

**Published:** 2023-12-15

**Authors:** Christopher Cole Honeycutt, Charles Griffin McDaniel, Autumn McKnite, J. Porter Hunt, Aviva Whelan, Danielle J. Green, Kevin M. Watt

**Affiliations:** 1 Duke University Durham North Carolina USA; 2 Department of Pharmacology and Toxicology, University of Utah College of Pharmacy Salt Lake City Utah USA; 3 Division of Clinical Pharmacology, Department of Pediatrics, University of Utah Medical Center Salt Lake City Utah USA; 4 Division of Critical Care, Department of Pediatrics, University of Utah Medical Center Salt Lake City Utah USA

**Keywords:** Extracorporeal membrane oxygenation, Continuous renal replacement therapy, Drug extraction, Meropenem, Pharmacokinetics

## Abstract

*Background*: Meropenem is a broad-spectrum carbapenem-type antibiotic commonly used to treat critically ill patients infected with extended-spectrum β-lactamase (ESBL)-producing Enterobacteriaceae. As many of these patients require extracorporeal membrane oxygenation (ECMO) and/or continuous renal replacement therapy (CRRT), it is important to understand how these extracorporeal life support circuits impact meropenem pharmacokinetics. Based on the physicochemical properties of meropenem, it is expected that ECMO circuits will minimally extract meropenem, while CRRT circuits will rapidly clear meropenem. The present study seeks to determine the extraction of meropenem from *ex vivo* ECMO and CRRT circuits and elucidate the contribution of different ECMO circuit components to extraction. *Methods*: Standard doses of meropenem were administered to three different configurations (*n* = 3 per configuration) of blood-primed *ex vivo* ECMO circuits and serial sampling was conducted over 24 h. Similarly, standard doses of meropenem were administered to CRRT circuits (*n* = 4) and serial sampling was conducted over 4 h. Meropenem was administered to separate tubes primed with circuit blood to serve as controls to account for drug degradation. Meropenem concentrations were quantified, and percent recovery was calculated for each sample. *Results*: Meropenem was cleared at a similar rate in ECMO circuits of different configurations (*n* = 3) and controls (*n* = 6), with mean (standard deviation) recovery at 24 h of 15.6% (12.9) in Complete circuits, 37.9% (8.3) in Oxygenator circuits, 47.1% (8.2) in Pump circuits, and 20.6% (20.6) in controls. In CRRT circuits (*n* = 4) meropenem was cleared rapidly compared with controls (*n* = 6) with a mean recovery at 2 h of 2.36% (1.44) in circuits and 93.0% (7.1) in controls. *Conclusion*: Meropenem is rapidly cleared by hemodiafiltration during CRRT. There is minimal adsorption of meropenem to ECMO circuit components; however, meropenem undergoes significant degradation and/or plasma metabolism at physiological conditions. These *ex vivo* findings will advise pharmacists and physicians on the appropriate dosing of meropenem.

## Introduction

Meropenem is a broad-spectrum carbapenem-type antibiotic that is routinely used as an empiric treatment of life-threatening infections in hospitalized adult and pediatric patients [1]. It is FDA-approved for the treatment of complicated skin, skin structure, and intra-abdominal infections [1]. It is also approved for the treatment of bacterial meningitis in pediatric patients 3 months of age and older [1]. Because of these indications, meropenem is often used in critically ill patients on extracorporeal life support (ECLS) such as extracorporeal membrane oxygenation (ECMO) or continuous renal replacement therapy (CRRT).

While ECLS can be lifesaving, mortality often exceeds 40% [2–6]. This high level of mortality is multifactorial and includes complications from the underlying critical illness (e.g., multi-organ failure) and direct complications from ECLS support (e.g., anticoagulation-related bleeding). In addition, some of this mortality may be attributed to suboptimal drug dosing, resulting from ECLS-induced changes in pharmacokinetics [7–10]. ECLS can influence pharmacokinetics via three general mechanisms: 1) Drug adsorption by circuit components; 2) Drug clearance by the circuit (e.g., hemofiltration and dialysis); and 3) Physiological alterations triggered by the circuit and/or underlying critical illness [9–16]. It is therefore important to understand how drugs, such as meropenem, interact with ECLS circuits.

During ECMO, nonspecific drug interactions with multiple circuit components, including the tubing, oxygenator, and hemofilter have been implicated in drug extraction from circulation [17]. High lipophilicity and high protein binding are associated with greater drug extraction in *ex vivo* ECMO studies [18, 19]. During CRRT, drug clearance by the hemofilter may be impacted by protein binding, volume of distribution, interaction with CRRT circuit components, and molecular weight [20]. CRRT preferentially removes drugs with low lipophilicity, low protein binding, and low molecular weight [21, 22].

Meropenem is a hydrophilic (logP −0.6) small molecule (383 Da) with low protein binding (~2%) and a low volume of distribution [17]. Based on these physicochemical properties, we hypothesized that meropenem would undergo rapid clearance by CRRT and minimal clearance by ECMO. However, previous studies done with older equipment present inconclusive data concerning meropenem extraction by ECMO and CRRT [23–32]. To address the incomplete understanding of how ECMO and CRRT impact the pharmacokinetics of meropenem, we designed a study to investigate meropenem extraction from *ex vivo* ECMO and CRRT circuits.

## Materials and methods

### Circuit configurations

**Figure 1:**
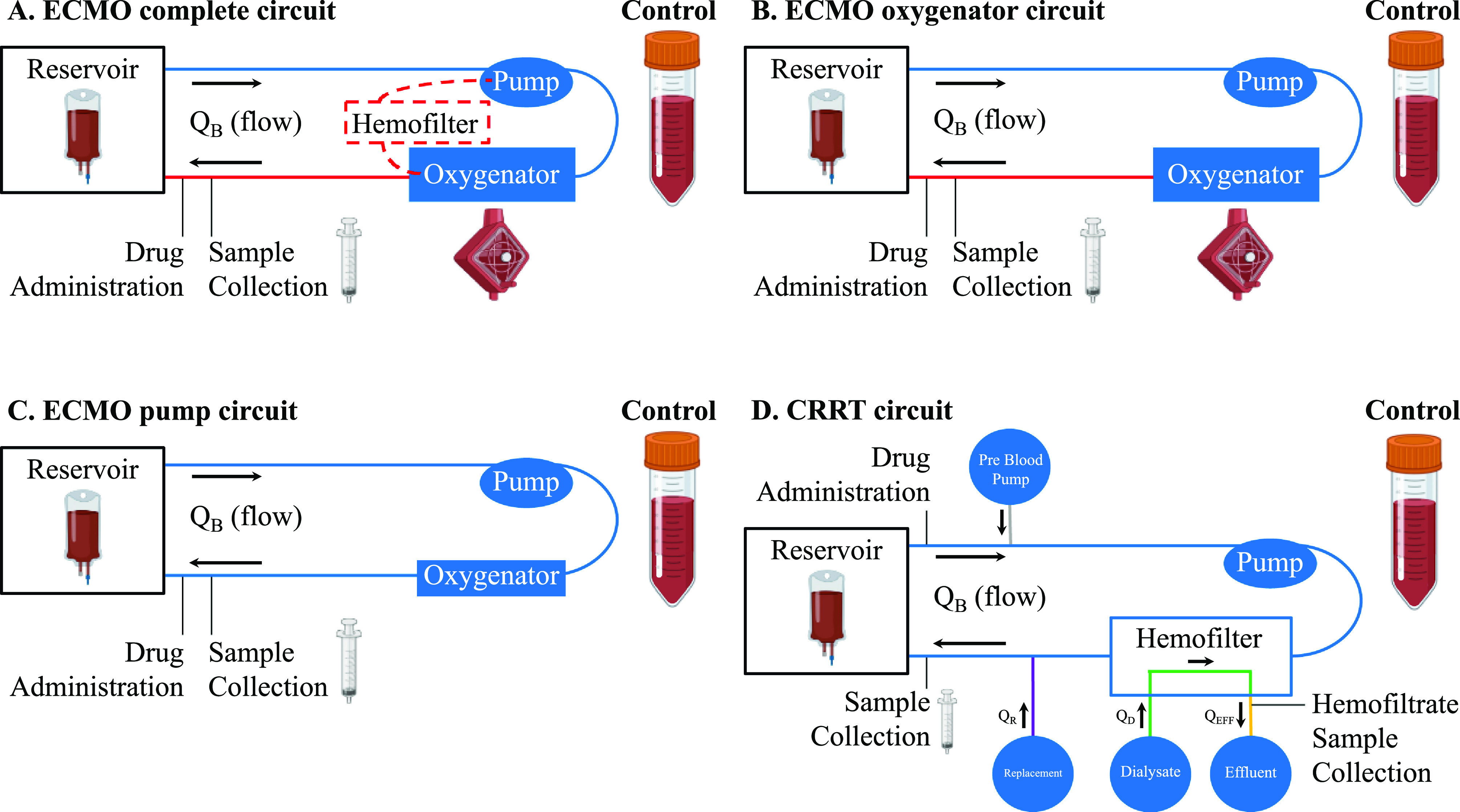
ECMO and CRRT circuit configurations. (A) ECMO complete circuit. (B) ECMO Oxygenator circuit. (C) ECMO Pump circuit. (D) CRRT circuit. ECMO – extracorporeal membrane oxygenation; CRRT – continuous renal replacement therapy.

To determine the contribution of each ECMO circuit component to drug extraction, three different ECMO circuit configurations were used (Figures 1A–1C). The ECMO Complete circuit included a 10-fr Bio-Medicus arterial cannula (Medtronic, Dublin, Ireland), 3/8-inch phosphorylcholine-coated polyvinyl chloride (PVC) Smart Tubing (Sorin, Saluggia, Italy), a Revolution centrifugal pump (Sorin), a DHF0.2 hemofilter (Sorin), and a Quadrox iD polymethylpentene adult oxygenator (Getinge, Gothenburg, Sweden) (Figure 1A). The ECMO Oxygenator circuit was identical, except that it lacked a hemofilter (Figure 1B). The ECMO Pump circuit lacked both a hemofilter and an oxygenator (Figure 1C).

CRRT circuits ran on a PRISMAX System (Baxter Healthcare) with a TherMax heater (Baxter Healthcare, Deerfield, IL) and HF1000 filter set (Baxter Healthcare, Deerfield, IL) connected via a 500 ml EXACTAMIX EVA bag (Baxter Healthcare, Deerfield, IL).

ECMO circuit configurations were run in triplicate and the CRRT circuit configuration was run in quadruplicate. One of four CRRT circuits failed following sampling at the 2-hour time point. Thus, CRRT circuit data includes three replicates with sampling out to four hours and one replicate with sampling out to 2 h.

### Extracorporeal membrane oxygenation circuit setup

ECMO circuits (Figures 1A–1C) were assembled according to standard clinical practice. The circuits were primed with a blood, plasma, and electrolyte mixture. The blood prime for the Complete and Oxygenator circuits consisted of 1 unit of packed human red blood cells (adenine saline added leukocytes reduced [~350 mL]), 0.5 units of human fresh human frozen plasma (~175 mL), and Plasma-Lyte A crystalloid (Baxter Healthcare, Deerfield, IL) (500 mL). In order to minimize the impact on clinical blood supply, we used recently expired blood products donated by the American Red Cross. Heparin sodium (500 units, 0.5 mL), sodium bicarbonate (7 mEq, 7 mL), tromethamine (2 g, 25 mL), calcium gluconate (650 mg, 6.5 mL), and albumin (12.5 g, 50 mL) were added to prevent coagulation and to mimic physiological conditions. In Pump circuits, all prime solution components added were scaled down to ⅔ of what was used in other circuit configurations because the oxygenator itself holds ~⅓ of the prime solution volume.

ECMO circuits were completed with a double-spiked intravenous bag, which had adequate volume to prevent air from entering. The flow was set to 1 L/min and measured post-oxygenator with an ultrasonic flowmeter (Sorin). In the Complete and Oxygenator circuits, a constant temperature of 37 °C was maintained with a Cincinnati Sub-Zero Hemotherm (Terumo Cardiovascular, Ann Arbor, MI). In the Pump circuit, a constant temperature of 37 °C was maintained via heating pads wrapped around the reservoir and tubing. Temperature and pH were monitored in real-time using a CDI Blood Parameter Monitoring System (Terumo Cardiovascular). Physiological pH (7.2–7.5) was maintained by the administration of sodium bicarbonate via the drug administration port and/or carbon dioxide via the sweep gas.

### Continuous renal replacement therapy circuit setup

CRRT circuits (Figure 1D) were assembled according to the manufacturer’s instructions for the HF1000 filter set. Circuits were primed with a solution of 1 unit of human red blood cells (adenine saline added leukocytes reduced [~300 mL]), ~0.4 units of human fresh human frozen plasma (125 mL), heparin sodium (350 units), sodium bicarbonate (7 mEq), tromethamine (1.5 g), calcium gluconate (180 mg), and human serum albumin (6.25 g). Blood was maintained at 37 °C by the TherMax blood warmer. Physiological pH (7.2–7.5) of circuit blood was tested each hour with an i-STAT 1 Analyzer (Flextronics Manufacturing, Singapore) and EG6+ cartridge (Abbott, Abbott Park, IL) and was maintained with tromethamine.

For pre-blood pump, dialysis, and replacement fluids, PrismaSATE 4/2.5 Dialysis Solution (Baxter Healthcare, Deerfield, IL) was used. CRRT circuits were run in continuous venovenous hemodiafiltration (CVVHDF) mode with the following specifications: blood flow rate (Q_B_) of 150 mL/min, dialysis fluid flow rate (Q_D_) of 1000 mL/h, pre-blood pump fluid flow rate of 700 mL/h, replacement fluid flow rate (Q_R_) of 300 mL/h delivered after filtration, and patient fluid removal net 0 mL/h. The 0 mL/h patient fluid removal setting induced the system to remove the extra fluid added by the pre-blood pump (700 mL/h) and replacement fluids (300 mL/h) via the effluent pump (Q_EFF_).

### Control setup

For ECMO and CRRT circuits, six controls were analyzed to determine drug degradation during the experiments. For these controls, 45 mL of blood prime solution was drawn from the primed circuit before drug administration but after at least 5 min of circulation to ensure adequate mixing and transferred to polypropylene centrifuge tubes (229,426, CELLTREAT, Pepperell, MA). The control samples were capped and maintained at 37 °C in a water bath.

### Drug administration and sample collection

Meropenem was dosed into the ECMO and CRRT circuits via arterial ports to achieve a concentration of 20 μg/mL and 50 μg/mL, respectively, both within the therapeutic range and above the minimum inhibitory concentration of meropenem [33–35]. The drug was administered at time = 0. The controls for ECMO and CRRT were also dosed to achieve concentrations of 20 μg/mL and 50 μg/mL, respectively. In controls, the drug was administered at time = −5 min. The test tubes were then capped and placed into a gentle rotator at room temperature. The control tubes were placed into a water bath at 37 °C at time = 0.

For ECMO circuits and controls, samples were collected at 1, 5, 15, and 30 min and 1, 2, 3, 4, 8, 12, and 24 h. For CRRT circuits and controls, samples were collected at 1, 5, 15, and 30 min and 1, 2, 3, and 4 h. In both ECMO and CRRT circuits, ~3 mL of circuit blood was drawn as “waste” with a syringe prior to sample collection and returned afterward. Samples were collected with syringes and then transferred to untreated microcentrifuge tubes. In CRRT circuits, hemofiltrate samples were collected at each time point just before the effluent bag. After sample collection in ECMO and CRRT circuits, the blood was centrifuged at 3,000 *g* for 10 min at 4 °C. Plasma was then pipetted into a cryovial (Fisher Scientific, Pittsburgh, PA) and stored at −80 °C. Hemofiltrate samples were transferred to a cryovial and stored at −80 °C after collection.

### Analysis

Meropenem concentrations in plasma and hemofiltrate were measured at OpAns Laboratory (Durham, NC) using high-performance liquid chromatography tandem mass spectrometry (HPLC-MS/MS) with [2H6] meropenem as an internal standard. Briefly, meropenem was extracted from 10 μL of a sample using methanol protein precipitation before analysis on an Agilent 6400 Series Triple Quadrupole Mass Spectrometer. Reverse phase chromatography using 0.1% (v/v) formic acid in water or methanol with a Poroshell 120 EC-C18 (Agilent) column preceded electrospray ionization in positive ion mode with multiple reaction monitoring (precursor and product ion m/z of 384/390 and 141/147 for meropenem and [2H6]-Meropenem, respectively). The assay was validated using standard curves achieving coefficients of determination (*R*^2^) > 0.9956 with coefficients of variation < 6.38% for concentrations across the range of the standard curves (50–100,000 ng/mL). The LLOQ for meropenem was 50 ng/mL and the accuracy ranged from 94.6% to 103.5%.

Due to differences in ECMO and CRRT circuit volumes, meropenem concentrations varied slightly between experiments. Drug recovery was therefore calculated using the following equation:

$$\mathrm{Recovery}\;(\%)\;=\;\frac{C_{t}}{C_{i}}\times 100,$$

where *C*_*t*_ is the concentration at time = *t* and *C*_*i*_ is the initial concentration. The initial concentration was set at time = 5 min, as there was inadequate mixing in ECMO and CRRT circuits at time = 1 min. Data are reported as mean and standard deviation (SD).

In CRRT, drug passage across the hemofilter was calculated from paired hemofiltrate and plasma samples at each time point using the following equation:

$$\mathrm{SA}\;=\;\frac{C_{H}}{C_{P}},$$

where SA is the saturation coefficient and *C*_*H*_ and *C*_*P*_ are concentrations in hemofiltrate and plasma, respectively.

Statistical tests for ECMO comparing all circuit configurations and control were performed at the 24-hour time point using one-way ANOVA. Statistical tests for CRRT comparing circuit and control were performed at the 2-hour time point using a paired t-test. GraphPad Prism Version 9.5.1 and Microsoft Excel were used for statistical analysis and graphing. Further details of statistical analysis and replicates are included in the figure legends. Lines represent the mean and error bars signify the standard deviation (SD). A supplementary table of all relevant raw data for ECMO and CRRT is available (Supplementary Table 1).

## Results

### ECMO circuits

**Figure 2:**
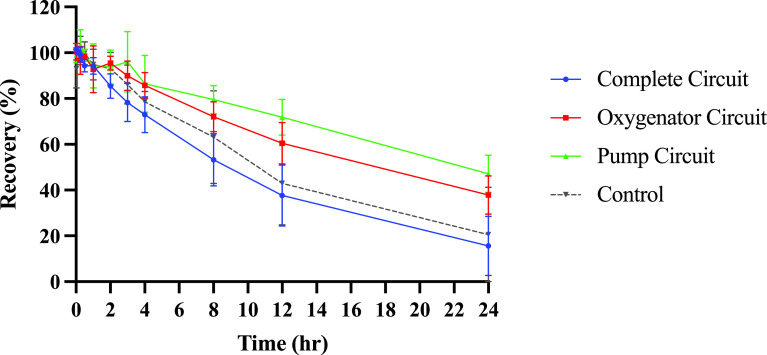
Recovery of meropenem in ECMO circuit configurations and controls over 24 h after administration. Values shown are means (*n* = 3) with error bars representing standard deviations. ECMO – extracorporeal membrane oxygenation.

All *ex vivo* ECMO circuit configurations and controls showed a steady decline in meropenem recovery over the course of 24 h (Figure 2). Mean (standard deviation, SD) recovery of meropenem at 24 hours was 15.6% (12.9) for Complete circuits, 37.9% (8.3) for Oxygenator circuits, 47.1% (8.2) for Pump circuits and 20.6% (20.6) for controls. At 24 h, there were no statistically significant differences in meropenem recovery between Complete, Oxygenator, and Pump circuits, and control (*p* = 0.0668). Two meropenem concentrations were not included in the analysis due to presumed contamination (Circuit 6 [Oxygenator Circuit] at *t* = 1 h; Circuit 9 [Pump Circuit] at *t* = 30 min).

### CRRT circuits

**Figure 3:**
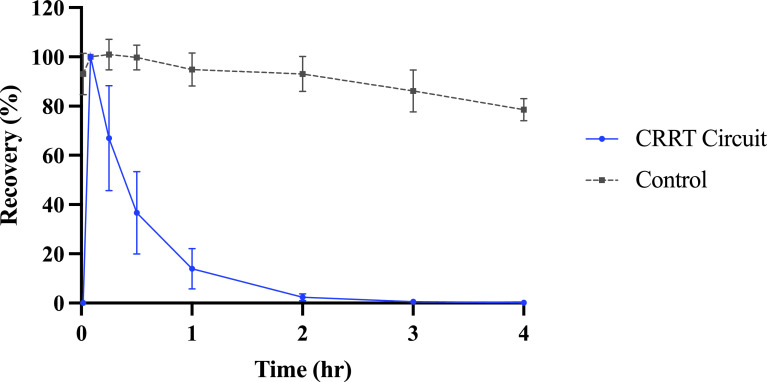
Recovery of meropenem in CRRT circuits and controls over 4 h after administration. Values shown are means (*n* = 4 for circuit, *n* = 3 for controls) with error bars representing standard deviations. CRRT – continuous renal replacement therapy.

Meropenem was rapidly cleared by CRRT with mean (standard deviation, SD) recoveries of 2.36% (1.44) at two hours and 0.13% (0.11) at 4 h in circuits compared with a mean (SD) recoveries of 93.0% (7.1) at two hours and 78.6% (4.5) at four hours in controls. The recovery in CRRT circuits was significantly different compared to the control at the two-hour time point (*p* < 0.0001) (Figure 3). The mean hemofiltration saturation coefficient (SA) was approximately one for the duration of the experiments, suggesting free filtering of meropenem from the circuits into the hemofiltrate. The average pH over ECMO and CRRT circuits was 7.37 (min = 7.12, max = 7.55). pH values outside of the target physiological range (7.2–7.5) were treated as per above.

## Discussion

ECMO and CRRT are critically important and lifesaving ECLS modalities. Many patients with severe infections, especially those caused by ​​extended-spectrum β-lactamase (ESBL)-producing Enterobacteriaceae, are treated with meropenem [1]. These patients may also be placed on ECMO and/or CRRT [36]. As there have been numerous reports of pharmacokinetic alterations and drug extraction in patients with ECLS, it is of interest to determine the extent of meropenem extraction in ECMO and CRRT. It is also of interest to determine the contributions of different circuit components to such drug extraction. In the present study, we demonstrate rapid extraction of meropenem from *ex vivo* CRRT circuits. We also saw substantial loss of meropenem in ECMO circuits but this loss was not significantly different from the controls suggesting that the loss is due to degradation rather than adsorption by the ECMO circuit. These findings were expected based on the physicochemical properties of meropenem.

Meropenem is a hydrophilic (logP −0.6) small molecule (383 Da) with low protein binding (~2%) and a low volume of distribution [17]. Prior studies have shown that these physicochemical properties predispose a drug to rapid clearance by CRRT dialysis and filtration and tend to limit adsorption to ECMO circuit materials [18, 19]. For CRRT, meropenem’s low molecular weight, high hydrophilicity, low volume of distribution, and low protein binding allow for free hemodiafiltration [20–22]. This was supported by our data that showed high meropenem recovery in the hemofiltrate, reflected by the mean saturation coefficient (SA) consistently around one. These factors suggest that meropenem can be rapidly cleared by CRRT and that clearance is related to CRRT flow rates [37]. These findings align with *in vivo* studies of critically ill patients with sepsis receiving CRRT, which have found that CRRT causes significant clearance of meropenem, necessitating steady-state intravenous doses of 500–1000 mg every 6–8 h to maintain sufficient plasma concentrations [30–32]. This is also consistent with results across β-lactam antibiotics, which as a class have target attainments that are highly impacted by RRT [24].

In the ECMO system, the loss of meropenem was not significantly different from the controls suggesting that degradation plays a major role rather than interaction with the ECMO circuit. This conclusion is supported by meropenem’s very short half-life, approximately 1 h [38]. Additionally, there is evidence that meropenem undergoes plasma metabolism. Studies of patients with end-stage renal disease and bilateral nephrectomy found that meropenem undergoes extrarenal metabolism or degradation with one detectable metabolite, the ring-open lactam form [39]. It has also been shown that the conversion of meropenem to the ring-open lactam form occurs at physiological pH and temperature but does not occur to a great degree at room temperature [39]. These findings have been observed in other *ex vivo* ECMO studies. Previous *ex vivo* work by Shekar et al. (2012) in isolated ECMO circuits identified a similar pattern of substantial loss in both circuits and controls [29]. However, in the Shekar study and a study by Cies, et al. (2022), there was a small but significantly greater loss in the circuits over time compared to controls suggesting some degree of interaction between meropenem and the circuit materials [23, 29]. Differences in equipment and PVC surface coatings used may help to explain the differences between our findings and the findings of these studies [23, 40].

*In vivo* pharmacokinetic studies of adult patients on ECMO have produced conflicting results. Shekar, et al. (2013) describe higher clearance of meropenem in ECMO patients compared with critically ill patients not on ECMO [26]. In contrast, a subsequent study by Shekar, et al. (2014) demonstrated a higher volume of distribution but lower clearance of meropenem in ECMO patients compared with critically ill patients not on ECMO [27]. Finally, Gijsen, et al. (2021) and Donatello et al. (2015) do not find significant differences in serum meropenem concentrations or target attainment between ECMO and non-ECMO patients [25, 28]. Given the minimal differences in *ex vivo* results, the conflicting *in vivo* results are likely due to patient-specific factors, such as differences in renal function between individual patients.

Our study has multiple limitations. First, while there are multiple different PVC surface coatings used currently in clinical practice, including heparin coating, we solely utilized phosphorylcholine-coated PVC tubing [23, 40]. Second, differences in the type of pump and diameter of the tubing could impact findings and were not explored in our study [23]. Third, our study design does not allow us to interrogate the roles of various additional mechanisms implicated in drug extraction from ECLS, including the ability of hemolysis to provide additional adsorption binding sites and release drugs from the cytoplasm of erythrocytes [41]. Fourth, our sample sizes were limited to controls (*n* = 3) and circuits (*n* = 3 for ECMO, *n* = 4 for CRRT). However, these sample sizes are consistent with previous *ex vivo* studies [9, 18, 29, 42]. Fifth, for CRRT, we did not investigate different effluent flow rates (Q_EFF_). For drugs that are cleared by hemofiltration or hemodialysis, the flow rate will impact the rate of clearance [37]. Because meropenem is a hydrophilic small molecule drug with low protein binding, we expect it to be freely filtered and thus impacted by fluid removal rate. In order to determine optimal dosing, future experiments that evaluate multiple fluid removal rates are necessary. Lastly, the results of *ex vivo* experiments, while modeling physiological conditions, do not recapitulate the physiological complexity of critically ill patients and are insufficient to guide optimal drug dosing recommendations. Factors including increased volume of distribution, altered drug clearance due to inflammation and other processes, and rapid changes in clinical condition (either improvements or deteriorations) contribute to the challenging problem of optimizing drug dosing in critically ill patients [43].

To address this, in the future, these data from *ex vivo* ECMO and CRRT circuits can be incorporated into physiologically based pharmacokinetic (PBPK) models, which integrate pharmacological and physiological data from critically ill patients to predict drug dosing requirements more accurately [44, 45]. In this manner, the data presented here concerning meropenem recovery and extraction can be utilized as a parameter to inform and improve PBPK models for critically ill patients concurrently on meropenem and ECLS. Follow-up clinical studies should additionally be conducted to confirm our *ex vivo* findings.

## Data Availability

The research data associated with this article are included within the article.

## Supplementary material

The supplementary material of this article is available at https://ject.edpsciences.org/10.1051/ject/2023035/olm *Supplemental Table 1*. Raw data from ECMO and CRRT circuits and controls.
